# Editorial: Naturalistic decision making (NDM): epistemic expertise in action

**DOI:** 10.3389/fpsyg.2023.1303098

**Published:** 2023-11-10

**Authors:** Julie Gore, Neil Shortland, Nicola Power, Olivia Brown

**Affiliations:** ^1^Department of Organizational Psychology, Birkbeck, University of London, London, United Kingdom; ^2^School of Criminology and Justice Studies, University of Massachusetts, Lowell, MA, United States; ^3^School of Management, University of Liverpool, Liverpool, United Kingdom; ^4^School of Management, University of Bath, Bath, United Kingdom

**Keywords:** epistemic expertise, naturalistic decision making, cognition, qualitative, cognitive task analysis (CTA)

Naturalistic decision making (NDM) is concerned with the study of how people make decisions and behave in complex, dynamic, and uncertain environments, in which decisions have significant consequences. NDM research is concerned with metacognitive expertise more broadly (e.g., sensemaking, perceptual skills), and applies methods such as cognitive task analysis to understand the unique aspects of decision-making under uncertainty. These processes are then use to design technologies and learning experiences for supporting individual and team performance in complex cognitive work systems. The epistemic development of NDM-related areas continues to grow and advance our understanding of complex decision-making in action.

The growing evidence base of epistemic NDM inquiry warrants that the impact of this field's innovative and rigorous task-based methods are documented for use and adoption by a larger audience of academics and practitioners. Traditionally NDM research has focussed upon high-reliability contexts and participants working in areas such as the military on land, air and sea, health and emergency services, challenging sporting decisions, intelligence, weather forecasting, fire fighting, trading, etc. often working with deep uncertainty and with complex problems completed by individuals and teams working within organizational constraints.

Whilst the NDM field is extremely well-known within the community of human factors and cognitive psychology scholars across the globe, its usefulness has received less attention across all organizational psychology-related contexts. The time is now ripe to share good research practice stories, perspectives and insights, and add value for practitioners and multi-disciplinary researchers across a more diverse range of organizations, to utilize the findings from NDM. By increasing our understanding of *why* and *how* experts see and handle decision challenges differently we can support and demystify associated myths and accelerate context, domain specific epistemic cognition (Brown et al., 2023).

The aim of this Research Topic is to share the value of the epistemic Naturalistic Decision Making (NDM) knowledge and research being conducted by cognitive psychologists and social scientists with a broad reach for practitioners. This work offers valuable methods and insights for all organizations who aim to elicit, document, and share expert knowledge of professionals at work.

The Research Topic opens with May et al. who provide an insightful systematic synthesis and holistic narrative analysis which identifies the challenges to critical incident decision making. Results suggested that research was moderately heterogeneous, research primarily focused toward intermediate meso-level characteristics, capturing factors such as “*interoperability*” and “*organization policy and procedure*” as critical challenges to decision-making. Six key narratives were identified and discussed. Six narratives that were thought to comprise decision-making for critical incident response emerged from the analysis: (1) political reform and modernization of emergency management doctrine; (2) difficulties of operating under austerity; (3) uncertainty and accountability; (4) inter-intra government and organizational ethics; (5) failures in collaborative information networks; and (6) limited research-focused horizon scanning. Both the quality appraisal and narrative findings suggested that research should seek opportunities to experimentally assess, evaluate and validate decision-making. Whilst this has previously appeared ethically and practically problematic, May et al. suggest that advances in technology, research and analysis have allowed high-fidelity simulation experimentation to recreate critical incidents.

Three original research contributions provide rich cognitive insights into: (i) security operations analysts (Reeves and Ashenden)—recommending an NDM framework to further understanding the challenges of this professional group; (ii) high performance coaches (Taylor et al.) who reveal that increasing adaptive skill is paramount to performance. Their findings suggest opportunities for utilizing Cognitive Task Analysis to investigate the cognitive challenges of sport coaching and enhance coach development practice. (iii) The adaptive performance of a drone, highlighting that decision-making can be an emergent capacity that arises from the interactions of both human and non-human agents in a socio-technical system (Kordoni et al.). Kordoni et al. investigate how human-robotic interactions can inform decision-making in emergency responders during mass-casualty events. Specifically, the authors explore whether it is possible to expedite the evacuation of casualties during an emergency by utilizing “identity-adaptive” drones in the process. The findings demonstrated how implementing a “identity adaptive robot” (i.e., a drone which is able to align conversationally with survivors) can aid in the evacuation efforts. This novel methodology provides promising evidence that future autonomous systems might be used to both alleviate the immediate evacuation of victims during major emergencies, whilst improving the situational awareness and reducing the cognitive load of responders.

Our Research Topic is then followed by two important Perspectives papers:

Papautsky applies NDM methods to provide a fascinating autoethnographic account in her paper “patient decision making in recovering from surgery.” Here she describes how the complex judgements faced by patients following surgery are as complex as those faced by professionals typically studied by NDM scholars. The main difference being that patients are not trained in how to make these judgements. She advocates that NDM methods offer a promising toolkit to develop understanding and inform support mechanisms to aid patient decision-making. Similarly, Dorton et al. argue that Naturalistic Decision Making (NDM) principles, models, and tools are well-suited to tackling the challenge that artificial intelligence (AI) developers foresee may mitigate harms that might result from their creations, noting that this is exceptionally difficult given the prevalence of emergent behaviors that occur when integrating AI into complex sociotechnical systems.

The final contribution to our Research Topic is by one of the leaders in the field of NDM, Klein et al. propose a plausibility transition model for sensemaking which has implications for the measurement and training of decision makers.

These articles illustrate how, after three decades, researchers continue to utilize the NDM framework, models and methods to explore complex cognition in uncertainty across a diverse range of professions and organizations. We would like to thank the authors, reviewers and Naturalistic Decision Making Association for their contributions, and we also wish to encourage those authors who didn't make it to publication this time, to be encouraged to continue to revise their manuscripts and engage with this pragmatic and diverse community of cognitive scholars and practitioners.

## Author contributions

JG: Conceptualization, Writing—original draft, Writing—review & editing. NS: Writing—review & editing. NP: Writing—original draft, Writing—review & editing. OB: Writing—original draft, Writing—review & editing.
